# Dietary soy and isoflavone intake and mortality in Korean adults: a prospective cohort study

**DOI:** 10.3389/fnut.2025.1613685

**Published:** 2025-07-09

**Authors:** Sihan Song, Shinyoung Jun, Hyojee Joung, Jung Eun Lee

**Affiliations:** ^1^Department of Food and Nutrition, College of Human Ecology, Seoul National University, Seoul, Republic of Korea; ^2^Division of Population Health Research, Department of Precision Medicine, National Institute of Health, Cheongju, Republic of Korea; ^3^Department of Food Science and Nutrition, Soonchunhyang University, Asan-si, Republic of Korea; ^4^Department of Public Health, Graduate School of Public Health, Seoul National University, Seoul, Republic of Korea; ^5^Research Institute of Human Ecology, Seoul National University, Seoul, Republic of Korea

**Keywords:** isoflavones, soy foods, mortality, cancer, cardiovascular disease

## Abstract

**Background:**

The association between dietary soy and isoflavone intake and mortality remains inconclusive. This study aimed to examine the relationships of dietary intakes of isoflavones, soy protein, and soy foods with all-cause, cancer, and cardiovascular disease (CVD) mortality in Korean adults.

**Methods:**

This prospective cohort study included 118,450 Korean adults aged 40–79 years from the Health Examinees Study (2004–2013). Dietary intakes of isoflavones, soy protein, and soy foods were assessed using a food frequency questionnaire. Cox proportional hazards models were used to estimate adjusted hazard ratios (HRs) and 95% confidence intervals (CIs) for mortality risk according to quartiles of dietary soy and isoflavone intake.

**Results:**

During a median follow-up of 10.1 years (interquartile range: 8.7–11.4 years), 2,614 deaths were documented, including 1,290 from cancer and 389 from CVD. Multivariable analyses showed no significant associations between dietary isoflavone intake and the risk of all-cause and cause-specific mortality. The HRs (95% CIs) comparing the highest vs. the lowest quartile of isoflavone intake were 1.04 (0.93–1.15) for all-cause mortality, 0.98 (0.84–1.14) for cancer mortality, and 1.04 (0.79–1.38) for CVD mortality. Similarly, no significant associations were observed for soy protein or soy food intake in relation to all-cause, cancer, and CVD mortality.

**Conclusion:**

Our study found no significant associations of dietary intakes of isoflavones, soy protein, and soy foods with the risks of all-cause, cancer, and CVD mortality.

## 1 Introduction

Soy foods are a major dietary source of isoflavones, plant-derived compounds classified as phytoestrogens due to their structural similarity to estrogens (1). Isoflavones are also considered natural selective estrogen receptor modulators (SERMs), exerting tissue-specific estrogenic or anti-estrogenic effects (2). Tamoxifen and raloxifene, for example, are widely used SERMs for the treatment of breast cancer and osteoporosis, respectively. In addition to their hormone-related actions, isoflavones have been reported to possess antioxidant, anti-inflammatory, anti-proliferative effects, and tyrosine kinase-inhibitory properties, which may contribute to a range of health outcomes (3). Soybeans also provide high-quality protein, which has been shown to exert a modest but clinically relevant cholesterol-lowering effect (4). Based on these biological properties, higher intakes of soy and isoflavones have been hypothesized to be associated with a reduced risk of coronary heart disease (CHD), certain cancers, improved bone health, and alleviation of menopausal symptoms such as hot flashes (5). However, findings from clinical and observational studies remain inconclusive across health outcomes, highlighting the need for further research to clarify the potential health effects of soy and isoflavones (5, 6).

Specifically, the relationship between dietary soy and isoflavone intake and mortality risk remains inconsistent (7–10). A recent meta-analysis of prospective cohort studies reported that the highest category of soy food intake, compared with the lowest, was significantly associated with lower risks of all-cause and cardiovascular disease (CVD) mortality, but not cancer mortality (8). In contrast, meta-analyses of isoflavone intake found no significant associations with mortality from CVD (9) or cancer (10). However, evidence on isoflavone intake and mortality risk is limited, particularly in populations with high habitual soy consumption. For example, in the Singapore Chinese Health Study, dietary intakes of soy protein, isoflavones, and tofu equivalents were not significantly associated with CVD mortality in the overall analysis (11). In sex-stratified analyses, higher soy protein intake was associated with a slightly increased risk of CVD mortality among men, but not women. In the Takayama Study, natto intake was inversely associated with CVD mortality among Japanese men and women, whereas no significant associations were found for soy protein or isoflavone intake (12). These limited and inconsistent findings underscore the need for further evidence from prospective studies conducted in populations with high habitual soy consumption.

Therefore, in this study, we aimed to examine the associations of dietary intakes of isoflavones, soy protein, and soy foods with all-cause, cancer, and CVD mortality in Korean adults, using data from a large-scale prospective cohort study.

## 2 Methods and materials

### 2.1 Study population

The Health Examinees (HEXA) Study is a prospective cohort study established as part of the Korean Genome and Epidemiology Study (KoGES) Consortium, conducted by the National Institute of Health within the Korea Disease Control and Prevention Agency (KDCA) (13). The HEXA study was designed to investigate risk factors for chronic diseases among Korean adults aged 40 years and older. Between 2004 and 2013, a total of 173,208 participants were recruited from 39 health examination centers, primarily general hospitals in metropolitan areas and major cities across Korea. Participants underwent an interview and health examination at baseline. For those who provided consent and had valid resident registration numbers, baseline data were linked to the Cause of Death Statistics provided by the Statistics Korea (14). Of the 130,230 participants in the linked dataset, individuals with a history of cancer or CVD (*n* = 8,664) were excluded. Participants who died within 3 years of follow-up (*n* = 474) were excluded to minimize potential reverse causation. Among the remaining participants, those without dietary data (*n* = 1,425) or with implausible energy intake (*n* = 1,217; beyond ± 3 standard deviations [SD] from the mean of log-transformed energy intake) were excluded, leaving 118,450 participants in the current analysis.

The HEXA study was approved by the Institutional Review Boards (IRBs) of the KoGES group collaborators and the KDCA. All participants provided written informed consent. This study received exempt approval from the IRB of Seoul National University (IRB No. E2012/001-002), and permission to use the data was granted by the National Institute of Health in Korea.

### 2.2 Dietary assessment

A validated semi-quantitative food frequency questionnaire (FFQ) with 106 food items was administered to participants through personal interviews (15). Participants were asked to report their average frequency of consumption for each food item over the past year, using nine frequency categories ranging from “almost never” to “three times per day.” They were also asked to report their usual portion size as small, medium, or large. Daily intake was calculated based on the selected portion size and frequency of consumption. The nutrient database for the FFQ was based on the food composition table of the Korean Nutrition Society (16).

### 2.3 Estimation of soy and isoflavone intake

Soy food intake (g/day) was calculated as the sum of the intake amounts of soybeans, tofu, soy sprouts, soy milk, and soybean paste (doenjang, cheonggukjang, and ssamjang). Soy protein intake (g/day) was calculated by summing the protein content of these soy foods. Total isoflavone content was calculated as the sum of daidzein, genistein, and glycitein, using the Flavonoid Database of Common Korean Foods (17). Each food item in the FFQ was matched to those in the isoflavone database, and dietary isoflavone intake (mg/day) was calculated by multiplying the amount of food intake by the isoflavone content. The percentage contribution of each food or food group to total isoflavone intake was calculated.

### 2.4 Ascertainment of mortality

Mortality data were obtained from the Cause of Death Statistics. The cause of death was categorized according to the Korean Standard Classification of Diseases, 6th revision, which is based on the International Classification of Diseases, 10th revision. Cause-specific mortality was classified into death from cancer (C00 to C97) and CVD (I00 to I99). The follow-up time for each participant was calculated from baseline to the date of death or the end of the follow-up period (December 31, 2019).

### 2.5 Covariate assessment

Data on demographics, lifestyle factors, and medical history were assessed using an interviewer-administered questionnaire (13). Average alcohol drinking over the past year was estimated as ethanol intake in grams per day, and smoking history was assessed in pack-years. Physical activity was measured by self-reported hours per week of regular exercise, defined as activity intense enough to cause sweating. Anthropometric measurements were obtained by trained staff, and body mass index (BMI) was calculated as weight (kg) divided by height squared (m^2^). Diabetes was defined based on a self-reported physician diagnosis. Participants also reported their regular use of multivitamins over the past year. Daily intake of total energy, fruits, vegetables, and red/processed meats was estimated from the FFQ.

### 2.6 Statistical analyses

Energy-adjusted intakes of isoflavones, soy protein, and soy foods were estimated using the residual method (18) and categorized into quartiles. All reported values for dietary soy and isoflavone intake were adjusted for energy intake. Cox proportional hazards models were applied to estimate hazard ratios (HRs) and 95% confidence intervals (CIs) for the association between dietary soy and isoflavone intake and mortality risk. Model 1 was stratified by 5- or 10-year age groups and adjusted for age in years and sex. Model 2 additionally adjusted for education level, current alcohol drinking (none, <5, 5 to <15, 15 to <30, ≥30 g/day), smoking status (none, <10, 10 to <20, 20 to <30, ≥ 30 pack-years), regular exercise [none, ≤ 3.5 (median), >3.5 hours/week], BMI (<18.5, 18.5 to <23, 23 to <25, ≥25 kg/m^2^), history of diabetes (no, yes), energy intake (kcal/day), and fruit and vegetable intake (g/day). Multivitamin use and red/processed meat intake were not included in the final model as they did not substantially affect the estimates. *P*-values for trend were calculated by entering the median value of each quartile of soy or isoflavone intake as a continuous variable in the model. The proportional hazards assumption was assessed using time-dependent covariates. Main analyses were repeated for men and women separately, using sex-specific quartiles. Subgroup analyses were conducted to examine the association between dietary soy isoflavone intake and all-cause mortality, stratified by demographic and lifestyle factors. Statistical significance for the interaction was tested using the likelihood ratio test.

A two-tailed *P*-value of < 0.05 was considered statistically significant. All analyses were performed using SAS version 9.4 (SAS Institute, Inc., Cary, NC, USA).

## 3 Results

### 3.1 Characteristics of participants

The median values (interquartile range) dietary intakes of isoflavones, soy protein, and soy foods were 10.2 (6.5–15.9) mg/day, 3.1 (2.0–5.1) g/day, and 34.8 (21.3–56.0) g/day, respectively. The major food sources of isoflavones were tofu (49%), soybeans (15%), soy milk (12%), soybean paste (11%), soybean sprouts (10%), with a combined contribution of 97%. Table 1 shows the baseline characteristics of participants according to quartiles of isoflavone intake. Participants with higher isoflavone intake were more likely to be older, women, engage in regular exercise, report a history of diabetes, and consume more fruits and vegetables.

**Table 1:** Baseline characteristics of Korean adults according to quartiles of dietary isoflavone intake.

	**Dietary isoflavone intake, mg/day**			
**Characteristics**	**Q1 (**<**6.5)**	**Q2 (6.5 to**<**10.2)**	**Q3 (10.2 to**<**15.9)**	**Q4 (**≥**15.9)**
*n*	29,612	29,613	29,613	29,612
Age, years	52.7 ± 8.3	52.4 ± 8.2	52.6 ± 8.2	53.1 ± 8.1
Women, %	61.0	63.4	65.9	70.6
Education level, %				
Elementary school or below	16.9	14.3	14.0	14.2
Middle school	15.9	15.0	15.4	15.6
High school	37.5	38.6	39.8	40.1
College or above	29.7	32.1	30.8	30.1
Alcohol drinking, g/day as ethanol	8.2 ± 30.3	8.0 ± 20.8	7.9 ± 22.3	7.0 ± 22.2
Smoking, pack-years	6.2 ± 13.1	5.7 ± 12.3	5.5 ± 12.0	4.7 ± 11.4
Regular exercise, hours/week	2.3 ± 3.7	2.6 ± 3.9	2.7 ± 3.9	3.0 ± 4.1
Body mass index, kg/m^2^	23.9 ± 2.9	23.9 ± 2.9	23.9 ± 2.9	23.8 ± 2.9
Self-reported diabetes, %	5.4	5.9	6.1	7.0
Multivitamin use, %	17.7	18.8	20.8	22.9
Energy, kcal/day	1,731 ± 493	1,743 ± 482	1,785 ± 517	1,745 ± 561
Fruits, g/day	166 ± 191	174 ± 170	191 ± 182	197 ± 191
Vegetables, g/day	237 ± 167	279 ± 164	320 ± 187	370 ± 244
Red/processed meat, g/day	44.8 ± 52.6	46.4 ± 47.7	47.5 ± 47.4	41.8 ± 46.3
Energy-adjusted intake				
Isoflavones, mg/day	4.5 ± 1.4	8.2 ± 1.1	12.8 ± 1.6	24.6 ± 10.3
Soy protein, g/day	1.4 ± 0.5	2.6 ± 0.6	4.2 ± 1.3	8.2 ± 3.7
Soy foods, g/day	14.5 ± 5.8	28.6 ± 7.0	45.5 ± 12.0	100.2 ± 64.7

Values are mean ± SD or percentage. *P*-values were calculated using the Kruskal-Wallis test for continuous variables and the chi-square test for categorical variables according to quartiles of dietary isoflavone intake. All variables had *P*-values < 0.001.

### 3.2 Dietary soy and isoflavone intake and mortality risk

During a median follow-up of 10.1 years (interquartile range: 8.7–11.4), 2,614 deaths were identified, including 1,290 attributed to cancer and 389 to CVD. Associations between dietary soy and isoflavone intake and mortality risk are presented in Table 2. In fully adjusted models, dietary isoflavone intake was not significantly associated with the risks of all-cause or cause-specific mortality. The HRs (95% CIs) for the highest quartile compared to the lowest quartile of isoflavone intake were 1.04 (0.93–1.15) for all-cause mortality, 0.98 (0.84–1.14) for cancer mortality, and 1.04 (0.79–1.38) for CVD mortality. Similarly, soy protein and soy food intake were not significantly associated with the risk of all-cause, cancer, or CVD mortality. When we conducted sex-specific analyses, no apparent association was observed in either sex (Table 3). The HRs (95% CIs) comparing extreme quartiles of isoflavones in men and women were 1.03 (0.90–1.19) and 1.03 (0.87–1.23) for all-cause mortality, 0.94 (0.76–1.16) and 1.06 (0.84–1.34) for cancer mortality, and 0.86 (0.60–1.24) and 1.21 (0.79–1.86) for CVD mortality.

**Table 2:** Hazard ratios and 95% confidence intervals for all-cause, cancer, and CVD mortality according to quartiles of dietary intakes of isoflavones, soy protein, and soy foods.

**HR (95% CI)**	**Q1**	**Q2**	**Q3**	**Q4**	***P* for trend**
**Isoflavone intake**					
Cut off, mg/day	<6.5	6.5 to <10.2	10.2 to <15.9	≥15.9	
Person-years	298,370	295,581	299,235	303,941	
**All-cause mortality**					
No. of deaths	687	607	643	677	
Model 1	Reference	0.97 (0.87–1.08)	0.99 (0.89–1.11)	1.00 (0.90–1.11)	0.81
Model 2	Reference	0.99 (0.89–1.10)	1.03 (0.92–1.15)	1.04 (0.93–1.15)	0.42
**Cancer mortality**					
No. of deaths	340	305	313	332	
Model 1	Reference	0.96 (0.82–1.12)	0.95 (0.81–1.10)	0.93 (0.80–1.09)	0.40
Model 2	Reference	0.97 (0.83–1.14)	0.97 (0.83–1.14)	0.98 (0.84–1.14)	0.84
**CVD mortality**					
No. of deaths	108	94	87	100	
Model 1	Reference	0.96 (0.73–1.27)	0.86 (0.65–1.14)	0.93 (0.71–1.23)	0.59
Model 2	Reference	1.01 (0.76–1.33)	0.93 (0.70–1.24)	1.04 (0.79–1.38)	0.81
**Soy protein intake**					
Cut off, g/day	<2.0	2.0 to <3.1	3.1 to <5.1	≥5.1	
Person-years	298,386	294,901	297,645	306,196	
**All-cause mortality**					
No. of deaths	672	605	632	705	
Model 1	Reference	0.95 (0.86–1.07)	0.98 (0.88–1.09)	0.98 (0.88–1.09)	0.94
Model 2	Reference	0.97 (0.87–1.08)	1.00 (0.90–1.12)	1.02 (0.91–1.13)	0.55
**Cancer mortality**					
No. of deaths	329	315	301	345	
Model 1	Reference	0.97 (0.83–1.13)	0.90 (0.77–1.05)	0.91 (0.78–1.06)	0.24
Model 2	Reference	1.01 (0.86–1.17)	0.94 (0.80–1.10)	0.98 (0.84–1.14)	0.72
**CVD mortality**					
No. of deaths	102	99	91	97	
Model 1	Reference	1.03 (0.78–1.36)	0.92 (0.69–1.22)	0.87 (0.66–1.15)	0.25
Model 2	Reference	1.07 (0.81–1.41)	0.99 (0.75–1.32)	0.97 (0.73–1.29)	0.68
**Soy food intake**					
Cut off, g/day	<21.3	21.3 to <34.8	34.8 to <56.0	≥56.0	
Person-years	298,414	297,129	300,044	301,541	
**All-cause mortality**					
No. of deaths	673	629	644	668	
Model 1	Reference	1.00 (0.89–1.11)	0.99 (0.89–1.11)	1.06 (0.95–1.18)	0.25
Model 2	Reference	1.02 (0.92–1.14)	1.03 (0.92–1.15)	1.10 (0.99–1.23)	0.08
**Cancer mortality**					
No. of deaths	331	318	315	326	
Model 1	Reference	1.01 (0.87–1.18)	0.97 (0.83–1.13)	0.98 (0.84–1.15)	0.75
Model 2	Reference	1.02 (0.88–1.19)	0.99 (0.85–1.16)	1.04 (0.89–1.21)	0.70
**CVD mortality**					
No. of deaths	115	84	91	99	
Model 1	Reference	0.78 (0.59–1.04)	0.83 (0.63–1.09)	0.92 (0.70–1.20)	0.85
Model 2	Reference	0.83 (0.63–1.10)	0.89 (0.68–1.18)	1.03 (0.78–1.35)	0.56

CVD, cardiovascular disease; HR, hazard ratio; CI, confidence interval. Model 1 was stratified by age (5 years for all-cause death, 10 years for cause-specific death) and adjusted for age in years and sex. Model 2 was additionally adjusted for education level (below elementary school, elementary school, middle school, high school, or above high school), current alcohol drinking (none, <5, 5 to <15, 15 to <30, or ≥30 g/day), smoking status (none, <10, 10 to <20, 20 to <30, or ≥ 30 pack-years), regular exercise (none, ≤ 3.5, >3.5 hours/week), body mass index (<18.5, 18.5 to <23, 23 to <25, ≥25 kg/m^2^), history of diabetes (no, yes), energy intake (kcal/day), and fruit and vegetable intake (g/day).

**Table 3:** Hazard ratios and 95% confidence intervals for all-cause, cancer, and CVD mortality according to sex-specific quartiles of dietary intakes of isoflavones, soy protein, and soy foods.

**Dietary intake quartiles**	**Person-years**	**All-cause mortality**		**Cancer mortality**		**CVD mortality**	
		**No. of deaths**	**HR (95% CI)**	**No. of deaths**	**HR (95% CI)**	**No. of deaths**	**HR (95% CI)**
**Isoflavone intake (mg/day)**							
Men (*n* = 41,174)							
Q1 (<6.1)	103,471	384	Reference	182	Reference	65	Reference
Q2 (6.1 to <9.4)	102,271	353	1.00 (0.86–1.15)	172	1.00 (0.81–1.23)	53	0.89 (0.62–1.29)
Q3 (9.4 to <14.7)	103,717	382	1.03 (0.89–1.19)	168	0.92 (0.74–1.13)	51	0.84 (0.58–1.22)
Q4 (≥14.7)	104,845	437	1.03 (0.90–1.19)	198	0.94 (0.76–1.16)	59	0.86 (0.60–1.24)
*P* for trend			0.58		0.48		0.47
Women (*n* = 77,276)							
Q1 (<6.7)	194,975	280	Reference	144	Reference	43	Reference
Q2 (6.7 to <10.6)	193,213	236	0.93 (0.78–1.11)	127	0.95 (0.75–1.20)	35	0.97 (0.62–1.52)
Q3 (10.6 to <16.5)	195,681	261	1.01 (0.86–1.20)	145	1.06 (0.84–1.33)	38	1.05 (0.68–1.64)
Q4 (≥16.5)	198,954	281	1.03 (0.87–1.23)	154	1.06 (0.84–1.34)	45	1.21 (0.79–1.86)
*P* for trend			0.46		0.44		0.30
**Soy protein intake (g/day)**							
Men (*n* = 41,174)							
Q1 (<1.8)	103,338	362	Reference	175	Reference	59	Reference
Q2 (1.8 to <2.8)	102,363	364	1.02 (0.88–1.18)	172	0.98 (0.79–1.21)	60	1.04 (0.72–1.49)
Q3 (2.8 to <4.6)	103,233	369	1.00 (0.86–1.15)	165	0.88 (0.71–1.09)	50	0.85 (0.58–1.24)
Q4 (≥4.6)	105,371	461	1.05 (0.91–1.21)	208	0.94 (0.77–1.16)	59	0.86 (0.60–1.25)
*P* for trend			0.45		0.60		0.33
Women (*n* = 77,276)							
Q1 (<2.1)	195,061	262	Reference	135	Reference	39	Reference
Q2 (2.1 to <3.2)	192,476	252	1.00 (0.84–1.19)	141	1.08 (0.85–1.36)	42	1.17 (0.76–1.82)
Q3 (3.2 to <5.3)	194,615	254	1.00 (0.84–1.19)	136	1.02 (0.80–1.29)	38	1.08 (0.69–1.69)
Q4 (≥5.3)	200,671	290	1.04 (0.88–1.23)	158	1.07 (0.85–1.36)	42	1.08 (0.70–1.69)
*P* for trend			0.61		0.66		0.91
**Soy food intake (g/day)**							
Men (*n* = 41,174)							
Q1 (<20.2)	103,394	386	Reference	184	Reference	66	Reference
Q2 (20.2 to <32.4)	102,835	373	1.06 (0.92–1.22)	183	1.06 (0.86–1.30)	48	0.80 (0.55–1.16)
Q3 (32.4 to <51.4)	104,116	368	0.98 (0.85–1.14)	160	0.87 (0.70–1.07)	55	0.88 (0.61–1.27)
Q4 (≥51.4)	103,961	429	1.09 (0.95–1.26)	193	0.98 (0.80–1.21)	59	0.92 (0.64–1.32)
*P* for trend			0.30		0.62		0.90
Women (*n* = 77,276)							
Q1 (<22.0)	195,148	269	Reference	138	Reference	45	Reference
Q2 (22.0 to <36.1)	194,137	250	1.02 (0.85–1.21)	137	1.06 (0.83–1.34)	33	0.86 (0.54–1.34)
Q3 (36.1 to <58.5)	196,008	260	1.05 (0.89–1.25)	145	1.11 (0.87–1.40)	39	1.04 (0.67–1.60)
Q4 (≥58.5)	197,529	279	1.10 (0.93–1.31)	150	1.11 (0.88–1.40)	44	1.17 (0.76–1.79)
*P* for trend			0.23		0.41		0.30

CVD, cardiovascular disease; HR, hazard ratio; CI, confidence interval. Model was stratified by age (5 years for all-cause death, 10 years for cause-specific death) and adjusted for age in years, education level (below elementary school, elementary school, middle school, high school, or above high school), current alcohol drinking (none, <5, 5 to <15, 15 to <30, or ≥30 g/day in men; none, <5, or ≥5 g/day in women), smoking status (none, <10, 10 to <20, 20 to <30, or ≥30 pack-years in men; never or ever in women), regular exercise (none, ≤ median, > median), body mass index (<18.5, 18.5 to <23, 23 to <25, ≥25 kg/m^2^), history of diabetes (no, yes), energy intake (kcal/day), and fruit and vegetable intake (g/day).

### 3.3 Subgroup analyses

**Figure 1:**
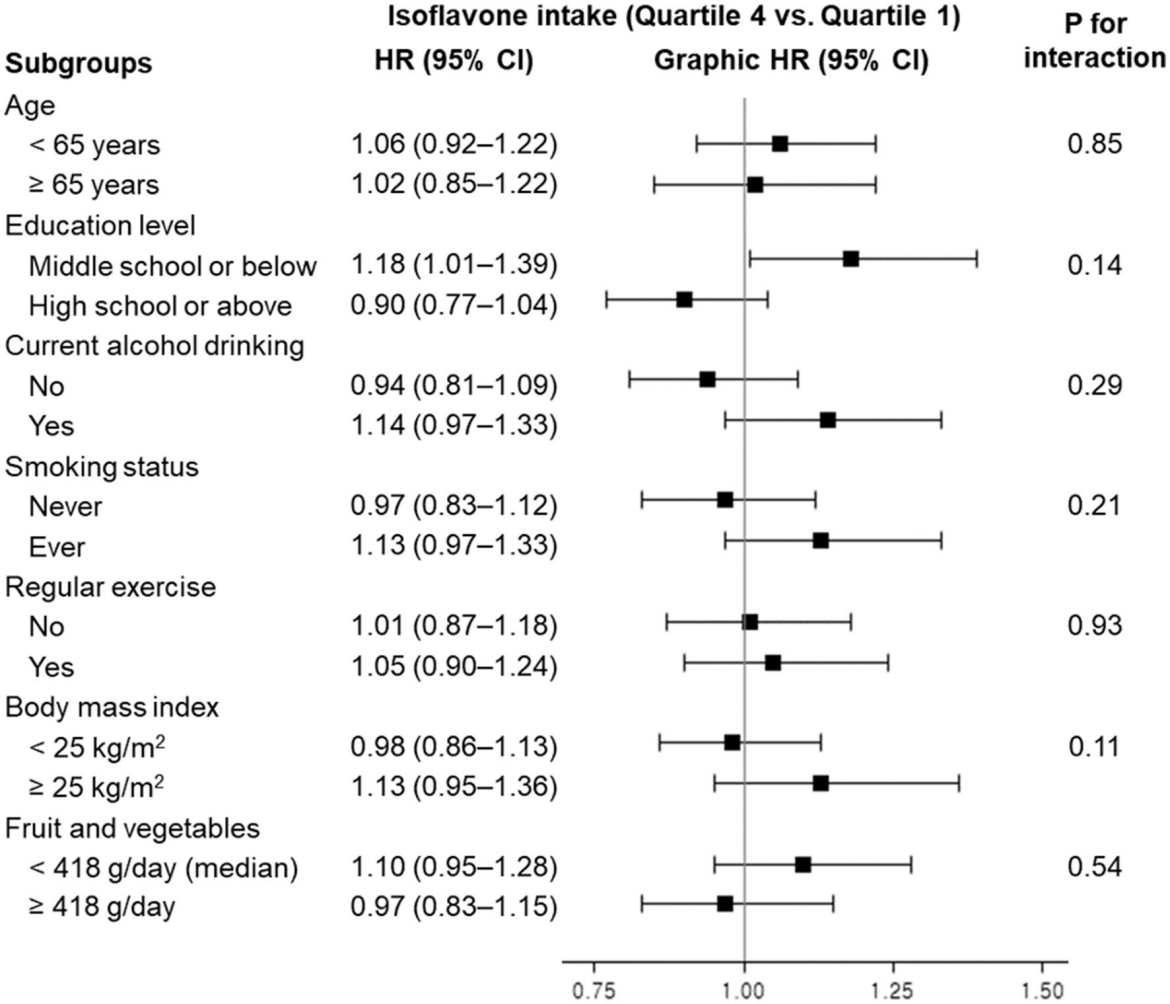
Hazard ratios and 95% confidence intervals for all-cause mortality according to quartiles of dietary isoflavone intake, stratified by demographic and lifestyle factors. HR, hazard ratio; CI, confidence interval. Model was stratified by age group and adjusted for age in years, sex, education level, current alcohol drinking, smoking status, regular exercise, body mass index, history of diabetes, energy intake, and fruit and vegetable intake (as applicable).

When we examined the association between dietary isoflavone intake and all-cause mortality, stratified by demographic and lifestyle factors, no significant interaction was observed (Figure 1). Although the interaction did not reach significance, higher isoflavone intake was associated with a slightly increased risk of all-cause mortality among those with an education level of middle school or below: HR (95% CI) comparing extreme quartiles was 1.18 (1.01–1.39).

## 4 Discussion

In this prospective cohort study of Korean adults, dietary intakes of isoflavones, soy protein, and soy foods did not appear to reduce the risks of all-cause, cancer, and CVD mortality.

Regarding all-cause mortality, most studies have shown an inverse association with isoflavone and/or soy food intake (19–24), but a few have not, particularly in studies where soy intake was low (25–27). No significant association was observed between dietary isoflavone intake and all-cause mortality risk among US postmenopausal women (25) and Spanish adults (26). Among Italian adults, higher isoflavone intake was associated with an increased risk of all-cause mortality (27). The median isoflavone intake in these studies was <1 mg/day. In US studies reporting significant inverse associations despite low isoflavone intake (median <1 mg/day), dose-response analyses demonstrated a linear association in men and women within the analyzed range (up to ~6 mg/day, excluding the top 2.5% of intake values) (23), and a non-linear pattern in women, with risk reduction plateauing around 6 mg/day and no additional benefit observed at higher levels (24). In our study, the cutoff for the lowest quartile of isoflavone intake was 6.5 mg/day, indicating substantially higher intake levels compared to populations with low soy food consumption. If threshold effects exist at very low intake levels, the habitual intake of soy foods in our population may partly explain the null findings. Additionally, among studies reporting significant associations between soy intake and mortality risk, several studies found no additional benefit at higher intake levels. The Guangzhou Biobank Cohort Study found that Chinese adults who consumed 1 to 6 portions of soy foods per week had a lower risk of all-cause mortality compared with those who consumed none, but no significant association was observed for those who consumed 7 or more portions per week (8). Similarly, a study in Hong Kong found comparable reductions in all-cause mortality risk in the third and fourth quartiles of soy food intake relative to the lowest quartile (21). In the Japan Public Health Centre-based Prospective Study, the reduction in all-cause mortality risk in the highest quintile of fermented soy intake was similar to that in the moderate quintiles, compared to the lowest quintile (22). In contrast, another Japanese study found that men who consumed soy either rarely or almost daily had higher risks of all-cause mortality than those who consumed soy 1–2 times per week, whereas no such association was observed among women (28).

Overall, we found no significant association between soy and isoflavone intake and mortality risk. However, in subgroup analyses, higher isoflavone intake was associated with a slightly increased risk of all-cause mortality among individuals with lower education levels. Given the prevalent consumption of soy foods in the Korean population, higher isoflavone intake in this subgroup might reflect differences in overall dietary patterns or diet quality, and residual confounding cannot be ruled out. Furthermore, inter-individual variability in isoflavone metabolism, particularly the equol producer phenotype, may have influenced the observed associations. Equol, a metabolite of daidzein produced by gut microbiota in only some individuals, is more prevalent in Asian populations (~50%−60%) than in Western populations (29). Considering its higher affinity for estrogen receptor-β compared to daidzein, equol has been hypothesized to enhance the biological activity of soy isoflavones (5, 29). Although the role of metabolic variability remains uncertain, it may have contributed to the null findings and highlights the need for further investigation.

In our overall and sex-specific analyses, no significant associations were found between dietary soy and isoflavone intake and the risk of mortality from cancer or CVD. Some studies have reported that higher intakes of soy and/or isoflavones are associated with a decreased risk of cancer (19, 21) or CVD (22, 23) mortality. Conversely, a few studies have also reported positive associations between isoflavone and soy food intake and prostate cancer mortality (30), and between soy protein intake and CVD mortality among men (11). Consistent with our study, meta-analyses of prospective cohort studies have reported null associations between soy and isoflavone intake and cancer mortality (8, 10), as well as between isoflavone intake and CVD mortality (9). A meta-analysis found that higher soy food intake was associated with a reduced risk of CVD mortality compared to lower intake (8). Moreover, significant inverse associations have been reported between soy and/or isoflavone intake and the risk of cancer (10) and CHD (9). The biological properties of soy and isoflavones, including their potential to reduce oxidative stress, inflammation, and low-density lipoprotein cholesterol levels, may contribute to their protective effects in various diseases (3, 4, 31, 32). The lack of association between soy and isoflavone intake and mortality in observational studies, including ours, may be partially explained by changes in dietary habits following the diagnosis of these diseases. Additionally, in our study, the relatively small number of CVD deaths may have limited the statistical power in sex-specific analyses. Although the associations were not statistically significant, we observed a modest trend toward lower CVD mortality with higher intakes of soy and isoflavones among men. Further investigation with repeated assessments of soy and isoflavone intake, relevant lifestyle factors, and longer follow-up is warranted to clarify their potential role in mortality risk.

Our study has several strengths, including a large sample size, long-term follow-up, and the use of registry-based mortality data. However, there are several limitations to consider. First, dietary intake was assessed only at baseline, and participants' dietary habits may have changed during the follow-up period. Second, measurement errors in dietary assessment, including recall bias, are inevitable. These limitations may have led to non-differential misclassification, which tends to attenuate associations toward the null. Third, the observational nature of the study limits our ability to infer causality, and unmeasured or residual confounding cannot be ruled out. To reduce the potential for reverse causation, participants with cancer or CVD at baseline or early follow-up were excluded. Fourth, the number of events in certain subgroups may have limited the statistical power to detect modest associations. Lastly, because participants were recruited from selected metropolitan areas in Korea, the generalizability of our findings may be somewhat limited.

## 5 Conclusion

In this prospective cohort study of Korean adults, we observed no significant associations between dietary soy and isoflavone intake and mortality from all causes, cancer, and cardiovascular disease. The potential threshold effects suggested in previous studies may not have been detectable in our population, possibly due to the relatively high habitual intake of soy. These findings highlight the need for further research incorporating repeated assessments of diet and lifestyle factors, along with consideration of individual variability in isoflavone metabolism, to better understand the complex relationship between soy intake and long-term health outcomes.

## Data Availability

The data analyzed in this study is subject to the following licenses/restrictions: the data that support the findings of this study are available with the permission of the National Institute of Health in Korea. Requests to access these datasets should be directed to the Clinical & Omics Data Archive (CODA) [https://coda.nih.go.kr/frt/index.do].
